# Visualizing Alternative Phosphorus Scenarios for Future Food Security

**DOI:** 10.3389/fnut.2016.00047

**Published:** 2016-10-28

**Authors:** Tina-Simone Neset, Dana Cordell, Steve Mohr, Froggi VanRiper, Stuart White

**Affiliations:** ^1^Department of Thematic Studies – Environmental Change, Centre for Climate Science and Policy Research, Linköping University, Linköping, Sweden; ^2^Institute for Sustainable Futures, University of Technology Sydney, Ultimo, NSW, Australia

**Keywords:** phosphorus sustainability, food security, future scenarios, systems approach, interactive visualization tool

## Abstract

The impact of global phosphorus scarcity on food security has increasingly been the focus of scientific studies over the past decade. However, systematic analyses of alternative futures for phosphorus supply and demand throughout the food system are still rare and provide limited inclusion of key stakeholders. Addressing global phosphorus scarcity requires an integrated approach exploring potential demand reduction as well as recycling opportunities. This implies recovering phosphorus from multiple sources, such as food waste, manure, and excreta, as well as exploring novel opportunities to reduce the long-term demand for phosphorus in food production such as changing diets. Presently, there is a lack of stakeholder and scientific consensus around priority measures. To therefore enable exploration of multiple pathways and facilitate a stakeholder dialog on the technical, behavioral, and institutional changes required to meet long-term future phosphorus demand, this paper introduces an interactive web-based tool, designed for visualizing global phosphorus scenarios in real time. The interactive global phosphorus scenario tool builds on several demand and supply side measures that can be selected and manipulated interactively by the user. It provides a platform to facilitate stakeholder dialog to plan for a soft landing and identify a suite of concrete priority options, such as investing in agricultural phosphorus use efficiency, or renewable fertilizers derived from phosphorus recovered from wastewater and food waste, to determine how phosphorus demand to meet future food security could be attained on a global scale in 2040 and 2070. This paper presents four example scenarios, including (1) the potential of full recovery of human excreta, (2) the challenge of a potential increase in non-food phosphorus demand, (3) the potential of decreased animal product consumption, and (4) the potential decrease in phosphorus demand from increased efficiency and yield gains in crop and livestock systems.

## Introduction

We are now in an unprecedented era of global environmental change with substantial known and unknown implications for humanity. Foresight, reevaluation of taken-for-granted assumptions, and careful planning are therefore required to ensure components of the “earth system” are maintained and sustained to support future generations. In a world that could be home to over nine billion people by the middle of this century, producing and securing access to sufficient food and other vital resources is likely to be a substantial challenge for humanity. Addressing such sustainability problems typically shrouded in complexity and uncertainty often requires the development of multiple and integrated scenarios, beyond narrowly forecasting of the current trajectory into the future (1).

### Global Phosphorus Scarcity: An Emerging Global Sustainability Challenge

Global phosphorus scarcity has increasingly been the focus of sustainability research for more than a decade and has expanded international attention from phosphorus as an environmental pollutant toward its critical and strategic importance for global food security (2–6). A growing body of literature addresses global phosphorus flows and phosphate rock reserves [e.g., Ref. (4, 5, 7–9)]. A number of regional, national, or local studies have addressed the specific implications of phosphorus demand and supply and provided valuable insight in the dynamics between the different sectors, namely the mining sector, agriculture, food industry, the waste and waste water sector as well as consumer perspectives (10–15). However, systematic analyses of alternative futures for phosphorus supply and demand throughout the food supply system that allows for analysis of diverse measures on an equal basis (e.g., comparing the phosphorus yield benefits of recycling manure versus changing diets) are rare. Many focus on phosphate rock supply and demand. Further, few studies have included key stakeholders as participants in future scenarios studies (16–18).

Phosphorus is an essential nutrient for all living organisms, including plants and animals (19–21). Since the mid-twentieth century, use of fertilizer derived from mined phosphate rock has increased significantly and has boosted crop yields and contributed to feeding billions of people (22). Fertilizers currently account for 82% of global phosphorus use and are expected to remain so in the near future (4, 23). An additional 8% of mined phosphate rock is used for livestock feed additives, and the remaining 10% serves other industrial uses, such as detergents, military applications, and food additives (24, 25).

Future global phosphorus availability is limited due to several factors. First, phosphate rock is a fossil resource, accumulated over hundreds of millions of years as sediments from the seabed or created by earth processes as magmatic occurrences (26). Globally, magmatic occurrences are fewer than sedimentary deposits, and often more costly to explore and develop into mines. The concentration of P_2_O_5_ is on average significantly higher in sedimentary deposits, which is a key factor determining the profitability of phosphate rock (27). The average grade has been in decline over the past few decades as the higher quality phosphate is mined first (6). Remaining reserves are also becoming more difficult to physically access, contain more impurities, generate more waste, require more energy per ton of P extracted, and hence costs are expected to increase.

Second, the geopolitics of global phosphate reserves has been the focus of recent debate in particular since the 800% phosphate price spike in 2008 (28) and the extensive increase of estimated reserves in 2010 (29), when the estimation of phosphate rock reserves in Morocco/Western Sahara increased from 5.7 to 50 Gt and total global reserves from 16 to 69 Gt (30). While reserve figures are still shrouded in uncertainty and lack of transparency (7), this drastic change in reserve data presented a more pronounced global imbalance, where Morocco/Western Sahara is now estimated to hold 72% of the global reserves, and the following 7 countries, China, Algeria, Syria, South Africa, Jordan, Russia, and the USA account for 20% of the global reserves (30). This development both highlights the geopolitical risks relating to so few countries controlling a resource that is indispensable for global food production and demonstrates the high uncertainty inherent in estimations of quantity and quality of global phosphate reserves.

As such, geopolitical or economical scarcity of phosphorus, in addition to physical scarcity (31), could result in increasing prices and supply disruptions. Gradual increase in phosphate fertilizer and commodity prices has been a long-term trend over the past decade (28, 32). If prices were to rise or spike further, this would affect the most vulnerable farming families first: already today close to one billion poor farmers and their families struggle to access fertilizer markets and put food on the table (33). If phosphate fertilizers are physically or economically inaccessible to farmers in the short- or long-term, this will in turn adversely affect global agricultural productivity and farmer livelihoods (34).

Other factors that create uncertainty about the future phosphorus supply are the future demand of phosphorus for food production, which is influenced by demographics and lifestyle changes such as changing diets (35), as well as the demand for biomass for energy production (36), and changing cropping patterns due to climate change (37). Exacerbating this is a lack of effective global governance to ensure phosphorus is available and accessible to all farmers, and to stimulate and support sustainable phosphorus measures such as recycling phosphorus and efficient use (38).

### A New Approach: Visualizing Future Pathways

In light of these dynamic preconditions and lack of transparent data on global phosphorus supply, it is evident that new approaches to this global sustainability challenge need to be considered, such as a specific focus on future pathways to secure a sustainable supply of phosphorus for future food production. The inherent complexity of the global food system necessitates a robust dialog between the disparate stakeholders to identify key opportunities and challenges for more efficient phosphorus use and supply and to decrease phosphorus demand within food production and related sectors. To support such a dialog, various techniques can be used, such as interactive systems analysis or companion modeling (39), to enable potential users to participate in the analysis by exploring and evaluating future pathways. The need for such decision support tools is the point of departure for the conceptual development and evaluation of an interactive visualization tool to support the exploration of global phosphorus scenarios.

The interactive global phosphorus scenario tool presented in this paper is aimed at a general scientific and expert audience (such as policy or industry stakeholders involved in fertilizer or food production and distribution, sanitation, or environmental management) to facilitate visualization-supported dialogs on sustainable phosphorus futures. It involves key indicators and parameters that have been identified in prior research (5, 16) and builds on a global phosphorus model that covers the global food system (i.e., global crop and animal production) and related phosphorus flows, food processing, and consumption, as well as waste management. The tool provides a real-time visualization-supported interface to the global model of phosphorus demand and supply developed by the authors (presented in Section “Global Phosphorus Scenario Model V1.1”) and enables users to select pathways for meeting future food demand and to explore the effects of their own assumptions when testing different scenarios. The inherent interactivity allows for direct and real-time feedback, as the effects of any modifications of an input assumption (see Table 1 for user interface variables) are instantly displayed on the overall supply and demand of phosphorus for global food production. As such, a dialog between stakeholders can include a number of supply side and demand side changes simultaneously and cover both a shorter and longer time frame.

**Table 1:** **User interface variables on the dashboard for 2040 and 2070**.

Driver type	Variable	Input format
General	Global phosphate rock production	Total amount (Mt P)
	Global population	Total number (people)
Demand	Per capita meat and dairy consumption	% relative to 2007
	Portion of agricultural land dedicated to each production type	Total %
	Portion of global phosphate rock used for agricultural applications	Total %
Efficiency	Crop phosphorus efficiency for each land use type	% relative to 2007
	Livestock phosphorus efficiency for each land use type	% relative to 2007
	Crop yield gains for each land use type	% relative to 2007
	Livestock yield gains for each land use type	% relative to 2007
	Per capita food waste (domestic)	% relative to 2007
	Per capita food waste (supply chain)	% relative to 2007
Recovery	Portion of human excreta recycled	% of total
	Food waste recovery (domestic)	% relative to 2007
	Food waste recovery (supply chain)	% relative to 2007
	Portion of global manure reused in agriculture	% of total
	Portion of crop residues reused in agriculture	% of total

The aim of this paper is to present the outline of the global phosphorus model as well as the structure and future opportunities of the interactive global scenario tool. A number of test case future scenarios are also presented based on average global data for phosphorus demand and supply.

## Visualization-Supported Global Phosphorus Scenarios

The interactive global phosphorus scenarios tool is participatory, visualized, and systematic. It also facilitates multiple pathways. Table 2 indicates the functions associated with these features of the scenario tool.

**Table 2:** **Features and associated functions of the interactive global phosphorus scenario tool**.

Feature	Function
Interactive, participatory	Tackles inherent uncertainty in the future phosphorus-food system Increases acceptance from stakeholders rather than scientists prefixing assumptions, i.e., maximizes credibility and saliency Explores implications in real time to support dialog and decision-making Facilitates stakeholder engagement
Visualized	Communicates and synthesizes implications of complex data interactions in real time to support dialog and decision-making
Systematic	Allows for analysis of diverse measures on an equal basis (e.g., comparing the phosphorus yield benefits of recycling manure versus changing diets) Ensures the analysis does not miss or bias some options over others
Multiple pathways, scenarios	Allows for exploration and comparison of possible and preferred future pathways

### Future Phosphorus Scenarios

Current global sustainability challenges tend to involve a high level of complexity and uncertainty and therefore often require the development of multiple and integrated future scenarios. Scenarios provide images of possible future situations, rather than an expected or probable future (40–45). Scenarios on global phosphorus demand and supply enable the exploration of future pathways for sustainable phosphorus management. These explorations are an effective way to derive useful information, even without comprehensive and transparent data on global phosphorus flows or scientific consensus on the longevity of phosphate supplies at the international level.

**Figure 1:**
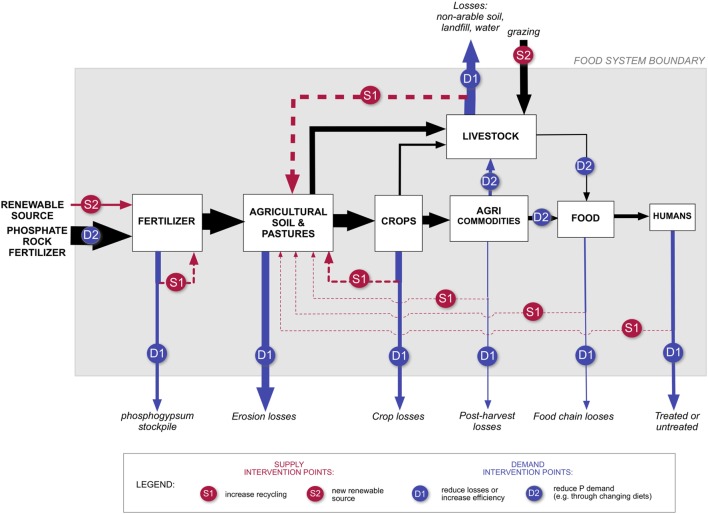
**The flow of phosphorus through the food system, indicating potential intervention points for phosphorus demand reduction (blue) and supply side interventions (red), such as increased recycling or renewable sources of phosphorus [From: Cordell and White (38)]**.

Previous sustainable long-term phosphorus scenario analysis, conducted by Cordell et al. (16), indicated a growing potential gap between the business as usual future demand for phosphorus to meet the world’s requirements for global food production and projected phosphate rock availability. Alternative futures were created according to probable, possible, and preferred futures and included a number of predefined assumptions that defined the potential change on the demand side – focusing on agricultural efficiency, human consumption, and losses throughout food production, storage, transport, processing, and distribution. These sustainable scenarios indicated that substantial infrastructural and institutional changes could decrease the future demand and increase alternative supply through an integrated approach involving efficiency measures in all sectors (mining and fertilizers, agriculture and livestock, food production and processing, food consumption), changing diets, and a high recovery rate of phosphorus from all sources: manure, human excreta, food waste, and crop residues (16, 38). Figure 1 indicates potential intervention points in the phosphorus system to achieve demand reductions and closed phosphorus loops through recycling.

A number of global phosphorus scenarios were presented by Van Vuuren et al. (18) and supported the expected increase of phosphorus consumption over the coming decades, for selected world scenarios. This increasing demand was followed by potential stabilization due to expected advances in feed additives, agricultural, and technological advances. The selected scenarios represented the Millennium Ecosystem Assessment scenarios and therefore provided four different alternative futures. While the fixed assumptions for alternative future scenarios are valuable for simplifying the analysis, they limit the exploration of other possible pathways or abrupt changes in pathways. Other phosphorus scenario studies covered future phosphorus scenarios for the European Union (11) or for specific parts of the food system (46, 47).

Some of the limitations of previous scenario studies, which often have a strong focus on specific supply trajectories, might be overcome by the interactive feature of the global scenario tool. This tool enables users to create their own scenario – independent of predefined storylines – and facilitates dialogs and unlimited model exploration. The rather generic nature of the interactive global phosphorus scenario tool entails certain limitations as well, in that it does not allow the specific exploration of detailed system information for specific regions or parts of the food system. For example, in countries like Japan and China, byproducts from the steel industry are a huge potential source of phosphorus (12).

### Visualization to Assist Science and Scenario Communication

Scenario modeling in general, and particularly within the fields of global change and sustainability sciences, often encounters specific challenges related to interpreting and communicating the inherent complexity. As with any research, the framework and results need to be communicated in a way that facilitates understanding and engagement despite the complexity of the model that lies behind the outputs. The integrated social–ecological nature of these fields demands high involvement and integration of different academic disciplines as well as sectors and policy areas. Highly interactive and flexible communication interfaces can assist in overcoming the challenge of involving experts and stakeholders in the process of validation and use of the scenario model. Vervoort et al. (48) identified a distinct lack of such communication efforts in scenario studies and points out the need for more interactive science and scenario communication.

Visualization, defined as a process by which an individual gains insight into complex systems (49), supports the generation of a mental image to allow humans to understand and interpret new information by revisiting, revising, and extending their internal representations. Visualization systems have the capacity to communicate information with greater speed and clarity (50). They provide an intuitive understanding for large and complex datasets and enable the inclusion and processing of more extensive content than through oral or written communication (49, 51). For data exploration and decision support, these are important features, as visualization-supported scenario tools have the potential to increase the engagement and encourage involvement in the deliberative process of the participants (48, 52–54).

Visual interfaces that apply advanced computer graphics have become more common in sustainability studies (55, 56) and can assist effective analysis, communication, and improved understanding of the implications of proposed policy measures, and to analyze the inherent complexity and uncertainty of future scenarios, either in a desktop or collaborative dialog setting.

The interactive global phosphorus scenario tool aims to facilitate stakeholder dialog to identify concrete priority options, such as investing in agricultural phosphorus use efficiency, or alternative renewable fertilizers derived from phosphorus recovered from wastewater and food waste. This first version was created to enhance the general understanding of the current situation as well as future opportunities and challenges for decreased demand and increased supply of phosphorus for food production. As such, it provides a flexible decision support tool for policy makers, industry, and other key stakeholders to explore different scenarios by selecting and changing various aspects of the phosphorus-food system.

## Global Phosphorus Scenario Model V1.1

### Model Framework

The formulation of the global phosphorus scenario model is described here by various components of the model. The model has a base year (2007) with historic actual data and performs all calculations on two future years: 2040 and 2070. The model determines estimates for other years in the time range 2007–2070 using cubic splines. For notational purposes, *t_c_* represents the base year (2007), and *t/t_c_* represents the comparison between the future year (*t* = 2040 or 2070) and the base year (*t_c_* = 2007). The methodology begins with phosphate rock supply in Section “Phosphate Rock Supply” and then outlines recycling supplies of phosphorus in Sections “Recycled Human Excreta,” “Recycled Domestic Food Waste,” “Recycled Supply Chain Wastes,” “Consumed Food,” “Livestock Land Required,” “Cropland for Livestock,” “Cropland Required for Human Consumption,” “Manure Recycled (from Livestock in Confinement),” and “Crop Residue Recycled.” In order to calculate manure recycling as well as phosphorus fertilizer demand, it is necessary to calculate the amount of land needed for farming (see Livestock Land Required, Cropland for Livestock, and Cropland Required for Human Consumption). Finally, the calculation of demand is presented in Section “Demand.”

#### Phosphate Rock Supply

The level of global dependence on phosphate rock supply is a key user input assumption. The user selects the level of phosphate rock supply in both 2040 and 2070.

#### Recycled Human Excreta

The amount of excreta recycled *S_E_*(*t*) is determined by Eq. 1:

(1) $$S_{E}\left(t\right)=r_{E}\left(t\right)p\left(t\right)E\left(t\right)$$

where *p*(*t*) is the global human population, which the user specifies for the two future years, *r_E_*(*t*) is the fraction of excreta recycled (user specified), and *E*(*t*) is the amount of excreta generated on average on a per capita basis determined by Eq. 2:

(2) $$E\left(t\right)=E\left(t_{c}\right)-\left(1-f_{M}\left(t/t_{c}\right)\right)\left(E\left(t_{c}\right)-E_{V}\right)$$

where *E_v_* denotes the average per capita *P* in the excreta of vegetarians (which is assumed to be constant over time), and *f_M_*(*t/t_c_*) denotes the per capita of animal-based food consumption (meat, eggs, dairy, etc.) in year *t* compared to the base year *t_c_* (the user can specify this fraction). Equation 2 is necessary as the average diet of the population influences the amount of phosphorus in the excreta; in particular, a higher meat diet can be linked to a higher phosphorus content in excreta.

#### Recycled Domestic Food Waste

The amount of domestic food waste recycled *S_W_*(*t*) is determined by Eq. 3:

(3) $$S_{W}\left(t\right)=r_{W}\left(t\right)F_{W}\left(t\right)$$

where *r_W_*(*t*) is the fraction of domestic food waste recycled (user specified value), and *F_W_*(*t*) is the amount of food waste generated (including preparation waste, spoilage, and inedible fractions), which is determined by Eq. 4:

(4) $$F_{W}\left(t\right)=f_{W}\left(t/t_{c}\right)F_{W}\left(t_{c}\right)\frac{p\left(t\right)}{p\left(t_{c}\right)}$$

where *f_w_*(*t/t_c_*) is the per capita amount of food wasted in year *t* compared to the base year *t_c_* [*f_w_*(*t/t_c_*) is a user selected value].

#### Recycled Supply Chain Wastes

The amount of food wastes in the supply chain recycled *S_S_*(*t*) is determined by Eq. 5:

(5) $$S_{S}\left(t\right)=r_{S}\left(t\right)F_{S}\left(t\right)$$

where *r_S_*(*t*) is the fraction of supply chain food waste recycled (user specified value), and *F_S_*(*t*) is the amount of waste food generated, which is determined by Eq. 6:

(6) $$F_{S}\left(t\right)=f_{S}\left(t/t_{c}\right)F_{S}\left(t_{c}\right)\frac{p\left(t\right)}{p\left(t_{c}\right)}$$

where *f_S_*(*t/t_c_*) is the per capita amount of food wasted in the supply chain in year *t* compared to the base year *t_c_* [*f_S_*(*t/t_c_*) is a user selected value].

#### Consumed Food

The amount of animal- and crop-based food physically consumed by the world’s population is denoted by *F_M_(t)* and *F_C_(t)*, respectively, and are determined by Eqs 7 and 8:

(7) $$F_{M}\left(t\right)=F_{M}\left(t_{c}\right)f_{M}\left(t/t_{c}\right)\frac{p\left(t\right)}{p\left(t_{c}\right)},$$

(8) $$F_{c}\left(t\right)=p\left(t\right)E\left(t\right)-F_{M}\left(t\right).$$

Equation 8 assumes that there is no accumulation of phosphorus in the human population.

#### Livestock Land Required

The livestock land is divided into three components: fertilized pastures, unfertilized pastures, and finishing areas. Estimating livestock land is necessary as finishing areas use crop foods to feed livestock, and fertilized pastures require phosphorus. The user inputs the fraction of livestock land that is dedicated to each farming type for the base and two future years. The fraction for livestock land using farming type *i* in year *t* is *h*_Mi_(*t*). *l_M_*(*t*) denotes the total amount of livestock land needed in year *t* to feed the world’s population. Hence, *l_M_*(*t*) *h*_Mi_(*t*) is the amount of livestock land dedicated to farming type *i* in year *t*. *L*_Mi_(*t*) denotes the total amount of livestock land needed to meet the world’s demand for animal products (taking into account losses), if all livestock land was of farming type *i*. If we assume a linear relationship between the size of livestock land *i* and the population that the land can support, then *l_M_*(*t*) *h*_Mi_(*t*) can support *l_M_*(*t*) *h*_Mi_(*t*)/*L*_Mi_(*t*) fraction of the world’s population. Since *l_M_*(*t*) is the amount of land needed to support the world’s population, we have:

(9) $$\sum_{i}\frac{l_{M}\left(t\right)h_{\text{Mi}}\left(t\right)}{L_{\text{Mi}}\left(t\right)}=1.$$

Rearranging this yields:

(10) $$l_{M}\left(t\right)=\frac{1}{\sum_{i}\frac{h_{\text{Mi}}\left(t\right)}{L_{\text{Mi}}\left(t\right)}}.$$

*L*_Mi_(*t*) is then calculated by:

(11) $$L_{\text{Mi}}\left(t\right)=\frac{f_{\text{Mi}}\left(t/t_{c}\right)p\left(t\right){\bar{L}}_{\text{Mi}}\left(t_{c}\right)}{1+A_{\text{Mi}}\left(t/t_{c}\right)}\left[\frac{F_{W}\left(t\right)+F_{C}\left(t\right)+F_{M}\left(t\right)}{F_{C}\left(t\right)+F_{M}\left(t\right)}\right]\times \left[\frac{F_{S}\left(t\right)+F_{C}\left(t\right)+F_{M}\left(t\right)}{F_{C}\left(t\right)+F_{M}\left(t\right)}\right]$$

where ${\bar{L}}_{\text{Mi}}\left(t_{c}\right)$ is the amount of livestock land of type *i* needed to feed one person (assuming no losses), and *A*_Mi_(*t/t_c_*) is the agricultural livestock efficiency gained between the base year *t_c_* and year *t* for farming type *i*. Equation 11 is complex but in essence has four components:

(1) The equation is governed by the change in meat consumption in the future year relative to the current year [*f*_Mi_(*t*/*t_c_*)] times population in the future year *p*(*t*) times the land needed to grow enough meat for one person in the current year ${\bar{L}}_{\text{Mi}}\left(t_{c}\right)$.
(2) The division by 1 + *A*_Mi_(*t/t_c_*) enables the calculation to take into account agriculture livestock efficiencies (which reduce the amount of land needed).
(3) The domestic food wastes [*F_W_*(*t*)] relative to food consumed [*F_C_*(*t*) + *F_M_*(*t*)] term in the first square bracket is increasing the amount of land needed to take into account domestic food waste.
(4) Similarly, the second square bracket term is increasing the land needed due to supply chain wastes.

#### Cropland for Livestock

It is assumed that only finishing areas require crop produce for feeding. It is also assumed that the cropland is entirely broadacre.^1^ The broadacre cropland required ${\hat{L}}_{\text{CB}}\left(t\right)$ is determined by

(12) $${\hat{L}}_{\text{CB}}\left(t\right)=L_{\text{CB/MF}}\left(t_{c}\right)l_{M}\left(t\right)h_{F}\left(t\right)\frac{1+A_{\text{MF}}\left(t/t_{c}\right)}{1+A_{\text{CB}}\left(t/t_{c}\right)}.$$

*L*_CB/MF_(*t_c_*) is the area of broadacre cropland needed to feed 1 km^2^ of finishing area livestock farming,^2^ which is multiplied by the finishing area *l_M_*(*t*)*h_F_*(*t*), and *A*_CB_(*t/t_c_*) is the agricultural cropland efficiency gained between the base year *t_c_* and year *t* for broadacre. An efficiency increase in finishing area farming results in more broadacre land needed, whereas efficiency increase in broadacre farming reduces the broadacre land needed.

#### Cropland Required for Human Consumption

This calculation is similar to livestock. First, there are three different cropland farming types: horticulture, broadacre, and “future compact farming” (see Data and Assumptions for explanations). The user inputs the fraction of cropland that is dedicated to each farming type for the base and two future years denoted as *h*_Ci_(*t*). Let *l_C_*(*t*) denote the total amount of cropland needed in year *t* to feed the world’s population and the finishing area animals. Then *l_C_*(*t*) *h*_Ci_(*t*) is the amount of cropland dedicated to farming type *i* in year *t*. Now, let *L*_Ci_(*t*) denote the total amount of cropland needed to feed the world’s populations’ crop needs (taking into account losses and excluding livestock consumption and ignoring feedlot animals requirements) if all cropland are of farming type *i*. If a linear relationship is assumed between the size of cropland i and the population that the land can support, then *l_C_*(*t*)*h*_Ci_(*t*) can support *l_C_*(*t*)*h*_Ci_(*t*)/*L*_Ci_(*t*) fraction of the world’s population and feedlot animals. Note $\frac{{\hat{L}}_{\text{CB}}\left(t\right)}{L_{\text{CB}}\left(t\right)}$ represents as a fraction the amount of broadacre cropland feedlot animals’ need relative to the amount of broadacre cropland the world’s population requires. Since *l_C_*(*t*) is the amount of land needed to support the world’s population and the feedlot animals:

(13) $$\sum_{i}\frac{l_{c}\left(t\right)h_{\text{Ci}}\left(t\right)}{L_{\text{Ci}}\left(t\right)}=1+\frac{{\hat{L}}_{\text{CB}}\left(t\right)}{L_{\text{CB}}\left(t\right)}.$$

With rearranging:

(14) $$l_{c}\left(t\right)=\frac{1+\frac{{\hat{L}}_{\text{CB}}\left(t\right)}{L_{\text{CB}}\left(t\right)}}{\sum_{i}\frac{h_{\text{Ci}}\left(t\right)}{L_{\text{Ci}}\left(t\right)}}.$$

*L*_Ci_(*t*) can be calculated by:

(15) $$L_{\text{Ci}}\left(t\right)=\frac{p\left(t\right)\left[{\bar{L}}_{\text{Ci}}\left(t_{c}\right)+\left({\bar{L}}_{\text{CiV}}\left(t_{c}\right)-{\bar{L}}_{\text{Ci}}\left(t_{c}\right)\right)\left(1-f_{M}\left(t/t_{c}\right)\right)\right]}{1+A_{\text{Ci}}\left(t/t_{c}\right)}\times \left[\frac{F_{W}\left(t\right)+F_{C}\left(t\right)+F_{M}\left(t\right)}{F_{C}\left(t\right)+F_{M}\left(t\right)}\right]+\left[\frac{F_{S}\left(t\right)+F_{C}\left(t\right)+F_{M}\left(t\right)}{F_{C}\left(t\right)+F_{M}\left(t\right)}\right]$$

where ${\bar{L}}_{\text{Ci}}\left(t\right)$ is the amount of cropland of type *i* needed to feed one person (assuming no losses on an average diet) in the base year, ${\bar{L}}_{\text{CiV}}$ is the amount of cropland of type i needed to feed a vegetarian (assuming no losses) in the base year, and *A*_Ci_(*t/t_c_*) is the agricultural cropland efficiency gained between the base year *t_c_* and year *t* for farming type *i*, which is selected by the user. This equation is similar in nature to Eq. 11, with the crop land needed essentially calculated as the population times the average persons crop land needs, where this amount changes depending on the diet, where a high meat diet requires more land than a low meat diet. Similarly, there are factors to control efficiency gains in farming and the supply chain and domestic food waste fractions.

#### Manure Recycled (from Livestock in Confinement)

The amount of manure recycled in year *t*, *S_M_*(*t*), is determined by Eq. 16:

(16) $$S_{M}\left(t\right)=r_{M}\left(t\right)M\left(t_{C}\right)\frac{l_{M}\left(t\right)h_{\text{MF}}\left(t\right)}{l_{M}\left(t_{c}\right)h_{\text{MF}}\left(t_{c}\right)}\left(1+A_{\text{MF}}\left(t/t_{c}\right)\right)$$

where *r_M_*(*t*) is the percentage of manure recycled (user selected input) and *M*(*t_c_*) is the amount of manure produced in the base year.

#### Crop Residue Recycled

The amount of crop residues recycled, *S_C_*(*t*), is determined by

(17) $$S_{C}\left(t\right)=r_{C}\left(t\right)C\left(t_{C}\right)\frac{F_{\text{TC}}\left(t\right)}{F_{\text{TC}}\left(t_{C}\right)}$$

where *r_c_*(*t*) is the fraction of residues recycled (user defined value). *C*(*t_c_*) is the crop residues generated in the base year, and *F*_TC_(*t*) is the total amount of crop-based foodstuff generated in year *t*. Now, *F*_TC_(*t*) is determined as the sum of crop food consumed by people, crop food wasted domestically, crop food wasted in the supply chain, and crop food fed to livestock. Mathematically, we can represent *F*_TC_(*t*) as

(18) $$F_{\text{TC}}\left(t\right)=F_{C}\left(t\right)+\frac{\left[F_{S}\left(t\right)+F_{W}\left(t\right)\right]F_{C}\left(t\right)}{F_{C}\left(t\right)+F_{M}\left(t\right)}+F_{C}\left(t\right)\frac{{\hat{L}}_{\text{CB}}\left(t\right)}{L_{\text{CB}}\left(t\right)}.$$

Equation 18, in words is total crop-based foodstuff = crop-based foodstuffs for humans assuming no losses plus crop-based food losses for humans plus crops needed for finishing areas livestock.

#### Demand

The demand for phosphorus is split into agriculture demand and other demand. The demand for phosphorus from cropland of type *i*, *D*_Ci_(*t*) is

(19) $$D_{\text{Ci}}\left(t\right)={\bar{D}}_{\text{Ci}}\left(t\right)l_{C}\left(t\right)h_{\text{Ci}}\left(t\right)\left(1-a_{\text{Ci}}\left(t/t_{C}\right)\right)$$

where ${\bar{D}}_{\text{Ci}}\left(t_{c}\right)$ is the amount of phosphorus needed to fertilize 1 km^2^ of cropland of type *i* in the base year, and *a*_Ci_(*t/t_c_*) is the assumed efficiency reductions in phosphorus applied in year *t* relative to year *t_c_* for farming type *i*. Similarly, the demand for phosphorus for livestock farming of type *i*, *D*_Mi_(*t*) is

(20) $$D_{\text{Mi}}\left(t\right)={\bar{D}}_{\text{Mi}}\left(t\right){\;l}_{M}\left(t\right){\;h}_{\text{Mi}}\left(t\right)\left(1-a_{\text{Mi}}\left(t/t_{C}\right)\right)$$

where ${\bar{D}}_{\text{Mi}}\left(t_{c}\right)$ is the amount of phosphorus needed to fertilize 1 km^2^ of livestock farming land of type *i* in the base year, and *a*_Mi_(*t/t_c_*) is the assumed efficiency reductions in phosphorus applied in year *t* relative to year *t_c_* for farming type *i*.

Finally, the demand for phosphorus from non-farming applications, *D_o_*(*t*), is determined by:

(21) $$D_{o}\left(t\right)=\frac{1-f_{A}}{f_{A}}\left(\sum_{i}D_{\text{Ci}}\left(t\right)+\sum_{i}D_{\text{Mi}}\left(t\right)\right)$$

where *f_A_* is the fraction of phosphorus demand associated with farming applications (user specified value).

### Data and Assumptions

Version 1.1 of the global phosphorus scenario model was created for selected years – the baseline year of 2007 and the two fixed years 2040 and 2070 for the future. These 2 years were selected since they were considered sufficiently far in the future to allow for substantial changes and innovations, yet not so far in the future as to be considered so unrealistic or abstract that they discourage users from engaging with the scenarios (57). For the baseline year, data were compiled based on a review of the literature. Baseline data for 2007 and associated parameters, sources, and assumptions are provided in Table S2 in Supplementary Material.

Key parameters of this system relate to land use and phosphorus use to support an individual consumer depending on

(1) the dietary pattern – portion of diet composed of vegetal products or animal products; and
(2) the type of production system:
  (i) crop production types are divided into
    (a) broadacre^3^
    (b) horticulture^4^
    (c) future compact farming^5^
  (ii) livestock production types are divided into
    (a) fertilized pasture^6^
    (b) unfertilized pasture
    (c) livestock in confinement^7^
    (d) fodder crops^8^

For horticulture and broadacre, an average global yield for the widest variety of crops was assessed and related to an average global fertilizer input for each of these crops. For the baseline data (future compact), current estimates for hydroponic crops were selected and assumed to provide the lower limit of productivity per area for future compact farming. These figures were used to calculate the area of land required to feed a specified diet in the model (see Supplementary Material).

With respect to livestock, as the above livestock types do not represent actual livestock farming (e.g., some are a combination in practice), these data were applied to the share of animal products attributable to each farming system type, over the course of their lifecycle. Each of these production systems has specified productivity levels that are expressed in units of land. To each such area, the average fertilization rate (for pasture or fodder crop) and amount of feed supplement are assigned.

This version focuses on the food system, hence excludes industrial use of phosphorus (e.g., detergents) and industrial sources of phosphorus (e.g., steel slag).

### The Visualization-Supported Interface

The interactive global phosphorus scenario tool provides a user-friendly interface with the model. It was specifically developed to enable phosphorus scenario exploration and information visualization. Focusing on the future supply and demand of phosphorus in food production, a requirement was that the interface in a comprehensive way represented changes for different scenarios and integrated the supply and demand curve in order to have a visual comparison of the deficit or surplus of supply in relation to the demand with inherent feedback mechanisms to ensure high user interactivity. As a general principle, the visualization interface had to meet a scientific standard of accuracy and representativeness, as well as legitimacy in terms of an adequate representation of data and uncertainties, to enable the user to follow the process and assumptions that are part of the model.

The interface was developed on the basis of the global phosphorus scenario model developed by the authors as described in Section “Model Framework” in collaboration with a Swedish visualization developer Infviz AB. The development has been an iterative process between the developer and the authors and involved a number of test sessions resulting in alterations. The first prototype (v1.0) was subjected to informal tests and evaluations with stakeholders and experts from academia, industry, and other organizations, and both form and content has been refined in accordance with the received feedback.

In order to facilitate the exploration of a variety of global phosphorus scenarios, the interface provides instant visual feedback to any changes made by the user during a session. The visualization tool follows a simple structure of two graphical displays and a selection of parameters that can be modified by the user.

**Figure 2:**
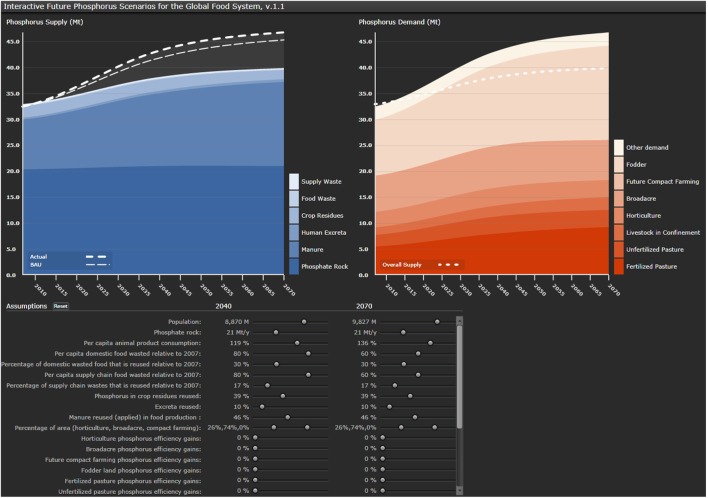
**Screenshot of the visual interface indicating phosphorus supply (left) broken down by various sources and demand (right) broken down by major agricultural land use types**. The dashed lines represent the “actual” (according to the selected scenario settings) versus the “BAU” (business as usual) demand. The opaque/gray area in the left graph indicates the “gap” between demand and supply.

The visual interface (Figure 2) is divided into a lower “dashboard” section where the selected parameter assumptions can be modified by users through a slide bar for the years 2040 and 2070. These user-activated parameters were selected based on higher associated uncertainty or controversy, to enable the user to test different hypotheses and to facilitate a discussion between groups of users on future options and alternatives. These parameters range from global population to the production of phosphate rock. The upper and lower limits were set in accordance with scientific literature, official statistics, or based on qualified assumptions where no ranges were obtained (see Supplementary Material for data sources and assumptions). For reasons of practicability and usability, a number of parameters were not included in the “dashboard” and therefore locked in the back end of the model – these were predominantly parameters that were either less controversial or that were related to data for the current situation rather than future trends. For each of the parameters, a default value is selected either as a mean value (e.g., mean scenario for population increase) or by the value given for 2007 [e.g., current rate (%) of reuse]. As the user modifies one or more of the parameters, the results for this changed scenario are represented simultaneously in the two graphical displays above, representing phosphorus supply and demand, both quantified in Mt of P (million tons of P). Results are not represented numerically in the scenario tool but visualized as a share of the overall demand and supply in the graphs.

The blue graph on the left represents the future phosphorus supply curve, which also integrates the results for the scenarios of future demand as dashed lines. These dashed lines indicate the future demand associated with different demand reductions – ranging from the business as usual scenario to the scenario where a number of sustainability choices such as changing diet and increased reuse and recycling have been implemented. The supply curve is divided into the different categories of phosphorus supply, namely, phosphate rock, manure, human excreta, crop residues, food waste, and supply waste. These are distinguished by different shades of blue and marked in a lateral legend.

The orange graph on the right represents the future phosphorus demand curve and integrates the total supply as a dashed line in accordance with the blue graph. The demand graph distinguishes different types of agricultural demand, namely, pasture, livestock in confinement, horticulture, broadacre, future compact farming, fodder, and other demand in shades of orange and identified in the lateral legend.

### Model Capability – Scenario Test Cases

**Figure 3:**
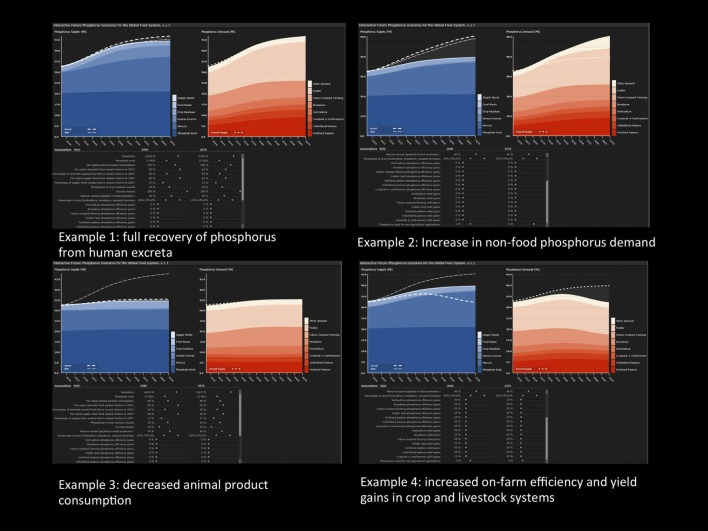
**Screenshot of test case examples: example 1: full recovery of phosphorus from human excreta, example 2: increase in non-food phosphorus demand, example 3: decreased animal product consumption, and example 4: increased efficiency and yield gains in crop and livestock systems**.

The interactive global phosphorus scenario tool is predominantly designed for a stakeholder user group, assisting the exploration of possible future pathways to discuss implications for sustainable resource management. For example, discussions could focus on the assessment of the global phosphorus demand for a significant demand side change such as consumption of animal products or an increase in efficiency in agriculture or food chain waste or supply side changes such as full recovery of phosphorus from human excreta. To demonstrate the capability of the tool, four examples are presented below and in Figure 3.

#### Example 1: Full Recovery of Phosphorus from Human Excreta

Increased recovery and reuse or recycling of human excreta has been proposed by many researchers and practitioners as one of several significant and to a large extent untapped phosphorus resource, with the potential to supply nutrients for food production where people live and grow their food (58, 59). As such, human excreta, in particular urine, which contains approximately 60% of excreted phosphorus, is the subject of a large body of research on recovery and reuse technologies as well as low-cost options to enable small-scale farmers to become independent of large external input of chemical fertilizer (60–63). In the current production system, however, the share of all human excreta remains only one-fifth of the total input of phosphorus through mined fertilizer to the system. To demonstrate this, the effect of 100% reuse of excreta is shown in Figure 3, where the full recovery of human excreta in 2040 and 2070 only provides a minor but nevertheless important source of phosphorus to the food production system.

This example could be supplemented by an assessment of other important recovery and reuse options (such as food waste recovery) or the total reduction of phosphorus use when combining with demand reduction options.

#### Example 2: Increase in Non-Food Phosphorus Demand

The demand for phosphorus in industrial applications (e.g., plastics, coatings, batteries) or for fertilizers for non-food products (e.g., biomass for energy production) at present only represents about 10% of the total mined phosphate rock (23). However, the possibility of a successive increase over the coming decades, due to changing policies or production priorities in particular in response to an increasing production of bioenergy (36), is a relevant scenario for exploration in the scenario tool. Therefore, example 2 focuses on an increase of other demands from 2.55 Mt P annually in 2007 to 3 Mt P in 2040 and 6 Mt P in 2070, indicating a doubled requirement of other uses, based on increasing global population and increased need for non-food production. This scenario could assist a discussion on future challenges in global land use and energy policies.

#### Example 3: Decreased Animal Product Consumption

The consumption of animal products accounts for a significant part of the total phosphorus input to the food system because phosphorus has to first pass through crop production before livestock production with associated inefficiencies in each stage [e.g., (35)]. A decrease in consumption of animal products would therefore influence the demand for phosphorus association with (a) fodder production, (b) fertilizers applied to pastures, and (c) phosphorus in animal feed supplements. Indirect system feedback loops are also important, for example, this decreased consumption of animal products would also decrease the available manure for phosphorus recovery and reduce the phosphorus concentration of human excreta and hence the total phosphorus available for reuse. To demonstrate this for the UN mean population increase scenario for 2040 and 2070 (64), a 20% decrease of the animal products consumption compared to the mean global consumption in 2007 is implemented. Other parameters in this example are kept in default setting, which influences the final result. Using this example as a real-time test case would require deciding on the setting of these other parameters prior to exploring changes in animal product consumption. For the specific selection criteria, as shown in Figure 3, this change would imply a decrease in total phosphorus demand and demonstrate a possible pathway to meet the global phosphorus supply. This example could inform a discussion on changing patterns of food consumption and the variety of related issues that need to be addressed in such a scenario, or contribute to an explorative exercise on identifying synergies between phosphorus management options and other sustainability challenges [e.g., (24)].

#### Example 4: Increased On-Farm Efficiency and Yield Gains in Crop and Livestock Systems

Fertilizer use efficiency in agriculture is expected to continue to increase in many regions (65) and is closely related to trends in global yield increases. Gregory and George (66) describe the linear global cereal yield improvement of 300% over 50 years, largely attributable to technological improvements. However, Grassini et al. (67) caution that the rate of global yield increase has decreased for major production areas and crops, calling for a careful assessment of future assumptions and projections on yield increase as they may be markedly lower than predicted. Even though the low efficiency of phosphorus use throughout the food system (25) leaves opportunities for improvement, example 4 focuses on modest assumptions of a 10% increase in 2040 and 20% increase in 2070 for all on-farm efficiency and yield gain parameters. For this scenario, the total phosphorus demand, would decrease below the given phosphorus supply threshold. Importantly, parameters linked to the production of animal products have the most significant impact to the overall demand of phosphorus. This scenario is similar to examples 1–3, based on default setting of other parameters that are not described here, and include multiple future assumptions that need to be addressed in a stakeholder discussion on alternative future pathways. The example could, however, be part of a practical assessment of potential future efficiency gains in nutrient use for food production.

The examples provided demonstrate various possible pathways, which the scenario tool could support in deliberative stakeholder processes. These examples should not be mistaken for results of future scenarios. Engaging in a proper assessment of future pathways would require a process of investigating all input parameters and assumptions or possible aims for the scenario. By manipulating single parameters, the user(s) can intuitively follow the impact various changes would have on the demand or supply of phosphorus and hence, the complexity of phosphorus in the global food system. The examples represent a selection of frequently discussed issues in sustainable phosphorus management and demonstrate how the tool could be used for interactive scenario exploration.

## Discussion

The interactive global phosphorus scenario tool provides a novel platform for scenario exploration and stakeholder involvement and enables a real-time high-level overview of the demand and supply side measures for sustainable phosphorus management to achieve global food security. It can be explored in a number of interactive settings, such as stakeholder workshops, decision-making forums, research workshops, and public communication forums. This first version, which is presented in this paper, was created to enhance the general understanding of the current situation as well as future opportunities and challenges for decreased demand and increased supply of phosphorus for food production. In contrast to traditional scenario studies, the model does not outline specific trajectories, but the interactive nature of this tool allows for the assessment of multiple pathways to close the gap between future phosphorus demand and supply.

As this version of the tool is limited to the global food production and consumption system, alternative approaches to phosphorus supply side management, such as recovery from the steel industry (12), are not included. Neither are alternatives like the mining of legacy phosphorus more specifically included (68). However, due to the global significance of these measures, they could be included in version 2 of the tool. Other measures might bear greater relevance in a national setup of the model. As the phosphorus supply and demand side management differs from one country to the other, a nationally appropriated tool would certainly be useful for cross-sectoral decision-making. We argue however, that the global phosphorus scenario tool as presented in this paper bears great potential to support dialogs between international stakeholders and to support the wider exploration of alternative futures, despite the inherent limitations that global average food production and consumption data and generic demand and supply side options imply.

The development of a national version of the tool, such as the Australian phosphorus scenario tool (69), allows both regionally specific parameters to be included and a measure of “people fed in current system” to account for imports and exports and the net surplus or deficit phosphorus, which is exported from the national food system as a mineral, food, or agricultural commodity. For multiple reasons, including data quality and availability, policy relevance, and the functionality as a resource management tool, national applications are considered an appropriate way forward, to complement the global tool.

A number of future improvements for v2.0 have been identified to enhance the usability and reliability of the tool. First and foremost is improved data, particularly for priority parameters, such as land area, feed, and animal production are required. Due to the generic character of this data assessment as well as its global scope, one of the inherent limitations of this model is data quality. The reliability of the fixed assumptions for the baseline year varies depending on the parameter and the number of reliable scientific sources on which the input value can be based. Uncertainty for the individual parameters, and for the scenario results in general, is exclusively communicated in a qualitative manner. Improved data can be enabled by an increased number of national studies as well as the growing body of literature on sustainable phosphorus management. The general idea for this scenario tool is to generate a platform of collaborative data validation and to improve its quality as the number of scientific studies in this field increases. Second, other globally significant measures outside the food system could be introduced, such as phosphorus recycling from industries such as the steel sector.

Third, the subsequent version of the tool will include explanatory pop-up boxes linked to each parameter, explaining the definition and significance each parameter or consequences of user input choices. Fourth, for future applications both usability studies in the form of stakeholder workshops and experimental studies are essential. Fifth, economic costs of selected recovery and efficiency measures and potential policy options would increase the relevance of the tool. Sixth, linkages to other resources or parameters, such as water, energy, or economic factors, could be included in the next model version, to enable the assessment of linkages between global phosphorus management and other global challenges, such as climate change, water scarcity, and energy supply (24, 70), as well as to identify what institutional and technical changes would be required to support a sustainable future supply and demand of phosphorus.

## Conclusion

Ultimately, this visualization-supported tool for interactive future phosphorus scenarios is designed to provide a platform to facilitate stakeholder dialogs and decision-making to identify concrete priority options, such as investing in agricultural phosphorus use efficiency or alternative renewable fertilizers derived from phosphorus recovered from wastewater and food waste. As such, it provides a flexible decision support tool for policy makers, industry, and other key stakeholders to explore different scenarios by allowing users to select and change various aspects of the phosphorus-food system to determine how phosphorus demand to meet future food security could be attained on a global scale in 2040 and 2070.

## Author Contributions

T-SN led the study, designed the scenario tool jointly with DC, and is the main author of the manuscript. DC designed the scenario tool jointly with T-SN, provided data for the modeling, and co-authored the paper. SM developed the model and contributed to the writing process. FV collected the data, and contributed to the development of the model and to the writing process. SW contributed to the scenario development, model development, and participated in the writing process.

## Conflict of Interest Statement

The authors declare that the research was conducted in the absence of any commercial or financial relationships that could be construed as a potential conflict of interest.

## Footnotes

^1^Crop production system for grain crops.

^2^The subscript *F* refers to finishing land, and subscript *B* refers to broadacre.

^3^Grain crops.

^4^Non-grain crops, including vegetables, fruits, roots, and pulses.

^5^Any efficient crop or food production system that has a high productivity per unit of land (typically technologically advanced, e.g., hydroponic crops grown in a controlled environment, using an aqueous solution or alternative substrate in place of soil, vertical gardens).

^6^Fertilized pasture refers to pasture that includes phosphorus fertilizer application.

^7^Animals fed in confinement or supplemented with harvested feed (excluding pasture-fed livestock).

^8^The average global mix of crops harvested for use as livestock feed.
