# Supplementary material for: Visualizing Alternative Phosphorus Scenarios for Future Food Security
Source: Front Nutr. 2016 Oct 28;3:47. doi: 10.3389/fnut.2016.00047 (PMC5083849; doi:10.3389/fnut.2016.00047)
Supplement: Supplementary file 1 [file Data_Sheet_1.PDF]

Supplementary Material

Table S1: P application rates for different agricultural production types - baseline data (2007), key parameters and assumptions

Parameter	Data	Assumptions & Sources	Inclusions/exclusions
Horticulture	3.71 tonnes/km^2 of P	Based on the global P_2O_5 application rate on major crop categories from FAO (2006), adjusted to assume 40% of global P applications are comprised by fertilizer, and 60% by manure (Potter et al., 2010).	Includes vegetables, fruits, roots and pulses
Broadacre	3.03 tonnes/km^2 of P	Based on the global P_2O_5 application rate on major crop categories from FAO (2006), adjusted to assume 40% of global P applications are comprised by fertilizer, and 60% by manure (Potter et al., 2010).	Includes grains
Future compact farming	13.1 tonnes/km^2 of P	A closed system with 100% P use efficiency was assumed. Figure based on average of P application rates for hydroponic wheat (Mackowiak et al., 1989), potatoes (Mackowiak et al., 1997), beetroot (Egilla, 2012), peppers (Majdi et al., 2012), and quinoa (Schlick and Bubenheim, 1996).	
Fodder crops	2.85 tonnes/km^2 of P	Total global hay production and P application rate from FAO (FAO, 2006; FAOSTAT, 2011) was combined with the annual portion of global production of coarse grains, wheat, oilseeds, and roots/tubers for fodder use from Hendy et al. (1995). For the latter crop categories, P application rates were based on FAO (2006).	Includes fodder crop blend
Fertilized pastures	1.64 tonnes/km^2 of P	Assuming 52,449,262 kg P applied to 924,631,000 ha, based on “fodder crop” P application rates from (FAO, 2007), applied to pasture areas for the reported countries, from (FAOSTAT, 2011)	

Table S2: Area required for different production categories - baseline data (2007), key parameters and assumptions[1]

Parameter	Data	Assumptions	Inclusions/exclusions
Area required to supply the annual caloric needs of a person - *100% Horticulture*	1 980 m^2/person (vegetarian)<br>1480 m^2/person (veg portion of omnivorous diet)	The average global yield for the widest variety of crop categories from FAOSTAT (2012a) was assessed, and the caloric value of this yield was calculated based on figures from Calorie Count (2012).	Horticulture
Area required to supply the annual caloric needs of a person - *100% Broadacre*	5 460 m^2/person (vegetarian)<br>4 220 m^2/person (veg portion of omnivorous diet)	As above	Broadacre
Area required to supply the annual caloric needs of a person - *100% future compact farming*	1.47 m^2/person (vegetarian)<br>1.10 m^2/person (veg portion of omnivorous diet)	Current yield estimates for hydroponic wheat (Mackowiak et al., 1989), potatoes (Mackowiak et al., 1997), beetroot (Egilla, 2012), peppers (Majdi et al., 2012), and quinoa (Schlick and Bubenheim, 1996) were averaged to create an approximate value for compact farming.	Future compact farming
Area required for - *Fertilized grazing*	2 190 m^2/person (Omnivore)<br>1 640 m^2/person (animal products portion of vegetarian diet)	Aggregated regional figures led to the assumption that the proportion of globally produced eggs, milk, beef, poultry, small ruminant meat, and pig meat attributable to harvested feed is 99.0%, 16.7%, 12.7%, 74.5%, 25.9%, and 99,0% respectively (based on global figures from (De Haan et al., 1997; Mphinyane, 2001; Nordblom et al., 1995)). These figures were applied to the FAO global production values for 2007 (FAOSTAT, 2012b).<br>Livestock production on fertilized and unfertilized pasture in 2007 was assumed at 2.3% and 97.7%, respectively (based on a comparison of fertilization rate (FAO, 2006) and total pasture area figures (FAOSTAT, 2011) from FAO), of the 36 200 000 km^2 global grazing land currently	to supply 22.2% of the annual caloric needs of a mixed diet through animal products fed exclusively on fertilized grazing<br>(i.e. assuming all animal products were produced via fertilized grazing)

[1] Land use for average global diet - The area required to feed a person in 2007 from any given production category was determined based on the assumption of a 2 450 kcal/day intake for 365 days, the middle range of global per capita food consumption reported by FAO (Bruinsma, 2003). The average global diet was assumed to comprise 77.1% vegetable matter and 17.0% farmed animal products, with the remaining intake made up by seafood (Bruinsma, 2003; Thornton 2010). The vegetal portion of the diet was assumed to comprise 45.4% horticulture products and 29.5% broadacre products, by calorie (Bruinsma, 2003). Seafood was assumed to consume neither land nor P fertilizer for purposes of this model.

		in use (R. P. White et al., 2000).	
Area required for - *Unfertilized grazing*	4 570 m^2/person (Omnivore)<br>3 423 m^2/person (animal products portion of vegetarian diet)	As above, assuming a 2.09x yield improvement of P-fertilized pastures over unfertilized pastures based on aggregated figures from (Bruinsma, 2003; Davison et al., 1997; Funderberg, 2007; Kopp et al., 2003; Robinson, D.L. et al., 2004)	to supply 22.2% of the annual caloric needs of a mixed diet through animal products fed exclusively on unfertilized grazing (i.e. assuming all animal products were produced via unfertilized grazing)
Area required for – *Livestock in confinement and fodder cropland*	9.59m^2/person (Omnivore)<br>7.18 m^2/person (animal products portion of vegetarian diet)	Determined based on aggregated figures from different regions of the world, comprising production values for eggs, milk, beef, poultry, and pig meat fed in confined areas, from (Carr, 1998; Cho and Kim, 2011; Deblitz and Reyes, 2012; European Union, 2007; French and Van Bysterveldt, 2011; J. P. Harner and Murphy, 1998; Henry and J. Harner, nd; Korir et al., 2010; Macdonald et al., 2001; Ministry of Agriculture, Gaborone, Botswana, n.d.; National Cattlemen's Beef Association, nd; National Chicken Council, 2012; Poultry Hub, 2012; Seidler, 2003; Thorne et al., 2009; Western Australia Dept of Agriculture, 2002; Zia et al., 2011).<br>Considered in the light of total global hay production from FAO (FAOSTAT, 2012a, 2011), and fodder crop production from Hendy et al. (1995), it can be assumed that at 10.9 m^2 cropland is required for each m^2 of finishing area.	to supply 22.2% of the annual caloric needs of a mixed diet through animal products attributable exclusively to harvested feed for finishing area<br>(i.e. assuming all animal products were produced via livestock in confinement)

Table S3: **Phosphorus supplements/ feed additives** - ***baseline data (2007), key parameters and assumptions***

**Parameter**	**Data**	**Assumptions**	**Inclusions/exclusions**
Livestock reared on fertilized pasture	0.132 t/km^2	Assuming 7% of global phosphate was used for feed additives (Potash Corp, 2012), and that this was evenly distributed across the total animal products produced in 2007[2].	
Livestock reared on unfertilized pasture	0.0631 t/km^2	As above	

[2] In reality, P supplements are only given to non-ruminant livestock (e.g. pigs, poultry).

Livestock reared in confinement	95.7 t/km^2	As above	

Table S4: **Waste types generated and recovered -** ***baseline data (2007), key parameters and assumptions***

**Parameter**	**Data**	**Assumptions**	**Inclusions/exclusions**
P in domestic food waste	0.476 Mt of P	Based on a sectorial and regional breakdown of food waste from FAO. (Gustavsson et al., 2011) and 2007 regional population data from the Population Reference Bureau (Haub, 2007), assuming that P comprises 0.52% of food waste dry weight (Zhang et al., 2005) and food waste contains 70% water.	
P in supply chain food waste	1.58 Mt of P	As above.	
Recovery rate – domestic food waste	30.3%	Average food waste recovery statistics and rural livestock ownership statistics for the major regions of the world were aggregated from several sources (Abou-Elseoud, 2008; Bengtsson and Sang-Arun, 2008; Cofie et al., 2006; Drechsel et al., 2007; Finpro Mexico, 2010; Furedy, 1990; Furedy et al., 1999; Harper, 2006; Khoo et al., 2010; Kido, 2011; McLeod, 2011; Meikle, 2009; Mwesigye et al., 2009; Refsgaard and Magnussen, 2009; US EPA, nd). Food waste recycling was assumed at 100% for livestock-owning families in developing countries.	include representative figures from both rural and urban environments
Recovery rate – supply chain food waste	17.0%	As above	As above
Excreta recycled in 2007	10%	Figure from (Cordell et al., 2009)	
P in crop residues (recoverable) in 2007	5.66 Mt P	Direct figure from (Potter et al., 2010).	
P in crop residues actually reused (returned to agriculture) in 2007	38.9%	9.6 Tg P/yr applied to agriculture in manures, out of 21.1 Tg P/year produced in manures (Potter et al., 2010).	

Total recoverable P in manure produced in 2007	21.1 Mt P	Assuming 3,758 Mt crop residues produced around the world (Lal, 2005), and P content of crop residues averaging 0.0015 kg/kg (Iqbal, 2009)	
The share of recoverable global manure that is being reused (applied) in food production	45.5%	Above assumption, considering 2.2 Mt P from crop residues reused on-farm annually (Smit et al., 2009).	

Table S5: Demographic and dietary data - baseline data (2007), key parameters and assumptions

**Parameter**	**Data**	**Assumptions**	**Inclusions/exclusions**
Global population	2007: 6,630 mil<br>2040: 8870 mil<br>2070: 9830 mil	Middle range population estimate and projections from (United Nations, 2010) were used	
Calories in average global diet	2,450 kcal/day	Calculated the middle range of global per capita food consumption from (FAO, 2003)	
Vegetable/animal product breakdown of global diet	77.1% of diet vegetables, 17.0% of diet animal products	Annual consumption (in kg) of cereals, roots and tubers, sugar, pulses, veg oils, meat, milk & dairy, fish, eggs, and “other” (assumed to be veg/fruit other than tubers & cereals) from FAO (Bruinsma, 2003) and Thornton (2010)).	excludes seafood
Per capita global P consumed – average diet	0.542 kg/person/yr	Assuming 98% of P in intake is excreted (Jönsson et al., 2004) and that the P content of adult diets averages 62 mg (2 mmol)/100 kcal in both sexes, (Institute of Medicine (US) Standing Committee on the Scientific Evaluation of Dietary Reference, 1997). Adjusted based on the following ratio: 847 mg P/2181 kcal meat diet; 818 mg P/2094 kcal vegetarian diet (Moe et al., 2011).	
Per capita global P consumed – vegetarian diet	0.545 kg/person/yr	As above	

Abou-Elseoud, N., 2008. Chapter 8: Waste Management, in: 2008 REPORT OF THE ARAB FORUM FOR ENVIRONMENT AND DEVELOPMENTArab Environment: Future Challenges.

Bengtsson, M., Sang-Arun, J., 2008. Chapter 6: Urban Organic Waste – From Hazard to Resource, in: H. Hamanaka, A. Morishima, H. Mori and P. King. Climate Change Policies in the Asia-Pacific: Re-uniting Climate Change and Sustainable Development. IGES White Paper. pp. 133–156.
Bruinsma, J. (Ed.), 2003. World agriculture: towards 2015/2030: an FAO perspective. Earthscan/James & James.
Calorie Count, 2012. Calori Count [WWW Document]. Calorie Count. URL http://caloriecount.about.com/ (accessed 11.25.12).
Carr, J., 1998. Garth pig stockmanship standards. 5M Enterprises.
Cho, J.H., Kim, I.H., 2011. Effect of stocking density on pig production. African Journal of Biotechnology 10, 13688–13692.
Cofie, O., Adam-Bradford, A., Dreschel, P., 2006. Recycling of Urban Organic Waste for Urban Agriculture, in: Van Veenhuizen, R. Cities Farming for the Future, Urban Agriculture for Green and Productive Cities. RUAF-IIRR-IDRC.
Cordell, D., Drangert, J.-O., White, S., 2009. The story of phosphorus: Global food security and food for thought. Global Environmental Change 19, 292–305.
Davison, T.M., Orr, W.N., Doogan, V., Moody, P., 1997. Phosphorus fertilizer for nitrogen fertilized dairy pastures. 2. Long term effects on milk production and a model of phosphorus flow. The Journal of Agricultural Science 129, 219–231.
De Haan, C., Steinfeld, H., Blackburn, H., Europea, U., 1997. Livestock & the environment: Finding a balance. European Commission Directorate-General for Development, Development Policy Sustainable Development and Natural Resources.
Deblitz, C., Reyes, E., 2012. Feedlots: A new tendency in global beef production? International Meat Secretariat Newsletter.
Drechsel, P., Graefe, S., Fink, M., 2007. Rural-urban food, nutrient and virtual water flows in selected West African cities (Research Report No. 115). Iwmi.
Egilla, J.N., 2012. YIELD AND LEAF ELEMENTAL CONCENTRATION OF BEETROOT IN RESPONSE TO NUTRIENT SOLUTION COMPOSITION IN HYDROPONIC CULTURE. Journal of Plant Nutrition 35, 203–214.
European Union, 2007. Council directive 2007/43/EC of 28 June 2007 laying down minimum rules for the protection of chickens kept for meat production. Off. J. Eur. Union 12, 19–182.
FAO, 2003. World agriculture: towards 2015/2030 - An FAO perspective - Ch 2: Prospects for food and nutrition.
FAO, 2006. FAO FERTILIZER AND PLANT NUTRITION BULLETIN 17: Fertilizer Use by Crop.
FAO, 2007. FertiStat Fertilizer Use Statistics Dataset.
FAOSTAT, 2011. Resources: Land [WWW Document]. FAOSTAT. URL http://faostat.fao.org/site/377/DesktopDefault.aspx?PageID=377#ancor (accessed 10.25.12).
FAOSTAT, 2012a. Production: Crops [WWW Document]. FAOSTAT. URL http://faostat.fao.org/site/567/DesktopDefault.aspx?PageID=567#ancor (accessed 10.23.12).
FAOSTAT, 2012b. Production: Livestock Primary [WWW Document]. FAOSTAT. URL http://faostat.fao.org/site/569/DesktopDefault.aspx?PageID=569#ancor (accessed 10.23.12).
Finpro Mexico, 2010. Waste Management in Latin America.
French, P., Van Bysterveldt, A., 2011. Infrastructural Requirements for a Greenfield Dairy Farm, in: Moorepark Dairy Levy Research Update, 15. Teagasc Greenfield Dairy Programme, Teagasc, Ireland.
Funderberg, E., 2007. Fertilizing winter pastures - Does it Pay? Ag News and Views.
Furedy, C., 1990. Social aspects of solid waste recovery in Asian cities. Environmental Sanitation Information Center, Asian Institute of Technology.
Furedy, C., Maclaren, V., Whitney, J., 1999. Reuse of Waste for Food Production in Asian Cities: Health and Economic. For Hunger-Proof Cities: Sustainable Urban Food Systems 136.
Gustavsson, J., Cederberg, C., Sonesson, U., Van Otterdijk, R., Meybeck, A., 2011. Global food losses and food waste. Food and Agriculture Organization of the United Nations, Rome.
Harner, J.P., Murphy, J.P., 1998. Planning Cattle Feedlots. Manhattan, KS: Kansas State University.
Harper, P., 2006. Feature article: Solid waste in Australia, in: Australia's Environment: Issues and Trends. Australian Bureau of Statistics.
Haub, C., 2007. 2007 World Population Data Sheet.

Hendy, C., Nolan, J., Leng, R., 1995. 3. FEED COMMODITY DEMAND AND SUPPLY, in: Interactions Between Livestock Production Systems and the Environment: Impact Domain: Concentrate Feed Demand, FAO Consultancy Report.

Henry, C., Harner, J., nd. Got Barnyard Runoff?

Institute of Medicine (US) Standing Committee on the Scientific Evaluation of Dietary Reference, 1997. Dietary reference intakes: for calcium, phosphorus, magnesium, vitamin D, and fluoride. National Academies Press.

Iqbal, S.M., 2009. Effect of Crop Residue Qualities on Decomposition Rates, Soil Phosphorus Dynamics and Plant Phosphorus Uptake. The University of Adelaide.

Jönsson, H., Stintzing, A.R., Vinnerås, B., Salomon, E., 2004. Guidelines on the Use of Urine and Faeces in Crop Production. EcoSanRes Programme.

Khoo, H.H., Lim, T.Z., Tan, R.B.H., 2010. Food waste conversion options in Singapore: Environmental impacts based on an LCA perspective. Science of The Total Environment 408, 1367–1373.

Kido, K., 2011. In Africa, producing food from waste. Christian Science Monitor.

Kopp, J.C., McCaughey, W.P., Wittenberg, K.M., 2003. Yield, quality and cost effectiveness of using fertilizer and/or alfalfa to improve meadow bromegrass pastures. Canadian Journal of Animal Science 83, 291–298.

Korir, M.K., Kibet, J., Kipsat, M.J., Nyangweso, P.M., Rirei, M., 2010. Dairy Cattle Productivity after the Post Election Crisis in Uasin Gishu District of Kenya, in: 2010 AAAE Third Conference/AEASA 48th Conference, September 19-23, 2010, Cape Town, South Africa.

Lal, R., 2005. World crop residues production and implications of its use as a biofuel. Environment International 31, 575–584.

Macdonald, K.A., Penno, J.W., Nicholas, P.K., Lile, J.A., Coulter, M., Lancaster, J.A.S., 2001. Farm systems-Impact of stocking rate on dairy farm efficiency, in: PROCEEDINGS OF THE CONFERENCE-NEW ZEALAND GRASSLAND ASSOCIATION. pp. 223–228.

Mackowiak, C.L., Owens, L.P., Hinkle, C.R., Prince, R.P., 1989. Continuous hydroponic wheat production using a recirculating system.

Mackowiak, C.L., Wheeler, R.M., Stutte, G.W., Yorio, N.C., Sager, J.C., 1997. Use of biologically reclaimed minerals for continuous hydroponic potato production in a CELSS. Advances in Space Research 20, 1815–1820.

Majdi, Y., Ahmadizadeh, M., Ebrahimi, R., 2012. Effect of Different Substrates on Growth Indices and Yield of Green Peppers at Hydroponic Cultivate. Current Research Journal of Biological Sciences 4, 496–499.

McLeod, A., 2011. Three human populations – three food security situations, in: World Livestock 2011-livestock in Food Security. Food and Agriculture Organization of the United Nations (FAO).

Meikle, J., 2009. The Middle East Is Drowning In Waste. Green Prophet.

Ministry of Agriculture, Gaborone, Botswana, n.d. APPENDIX 2: LAND REQUIREMENT FOR AGRICULTURAL ENTERPRISES: GUIDELINES.

Moe, S.M., Zidehsarai, M.P., Chambers, M.A., Jackman, L.A., Radcliffe, J.S., Trevino, L.L., Donahue, S.E., Asplin, J.R., 2011. Vegetarian compared with meat dietary protein source and phosphorus homeostasis in chronic kidney disease. Clinical Journal of the American Society of Nephrology 6, 257–264.

Mphinyane, W.N., 2001. Influence of livestock grazing within piospheres under free range and controlled conditions in Botswana. University of Pretoria.

Mwesigye, P., Batmale, J., Nyakang'o, J., Idan, I.A., Kapindula, D., Hassan, S., Van Berkel, R., 2009. Africa review report on waste management. Integrated Assessment of Present Status of environmentally-sound management of wastes in Africa', Prepared for UNIDO.

National Cattlemen's Beef Association, nd. FACT SHEET: Feedlot Finishing Cattle.

National Chicken Council, 2012. Animal Welfare for Broiler Chickens [WWW Document]. Industry Issues > Animal Welfare for Broiler Chickens. URL http://www.nationalchickencouncil.org/industry-issues/animal-welfare-for-broiler-chickens/ (accessed 11.24.12).

Nordblom, T.L., Goodchild, A.V., Shomo, F., 1995. Livestock, feeds and mixed farming systems in West Asia and North Africa. Livestock development strategies for low income countries.

Potash Corp, 2012. Markets & Industries: Feed [WWW Document]. URL http://www.potashcorp.com/investors/markets_industries/feed/ (accessed 11.24.12).

Potter, P., Ramankutty, N., Bennett, E.M., Donner, S.D., 2010. Characterizing the spatial patterns of global fertilizer application and manure production. Earth Interactions 14, 1–22.

Poultry Hub, 2012. Meat chicken farm sequence [WWW Document]. PRODUCTION > INDUSTRY STRUCTURE AND ORGANISATIONS > CHICKEN MEAT (BROILER) INDUSTRY. URL http://www.poultryhub.org/production/industry-structure-and-organisations/chicken-meat/meat-chicken-farm-sequence/ (accessed 11.24.12).

Refsgaard, K., Magnussen, K., 2009. Household behaviour and attitudes with respect to recycling food waste–experiences from focus groups. Journal of Environmental management 90, 760–771.

Robinson, D.L., Pinchak, W.E., Sij, J.W., Bevers, S. J., Gill, R.J., Malinowski, D.P., Baughman, T.A., 2004. Phosphorus Fertilizer Impacts Forage, Beef, and Grain Production from Wheat. Better Crops 88.

Schlick, G., Bubenheim, D.L., 1996. Quinoa: candidate crop for NASA's controlled ecological life support systems. Progress in New Crops. ASHS press. Arlington. VA 632–640.

Seidler, E.S., 2003. Chapter 1 - Egg production, in: Egg Marketing. A Guide for the Production and Sale of Eggs. FAO.

Smit, A.L., Bindraban, P.S., Schröder, J.J., Conijn, J.G., Van der Meer, H.G., 2009. Phosphorus in agriculture: global resources, trends and developments. Report to the Steering Committee Technology Assessment of the Ministry of Agriculture, The Neetherlands, Wageningen.

Thorne, P.J., Hengsdijk, H., Janssen, S., Louhichi, K., Van Keulen, H., Thornton, P.K., 2009. Modelling livestock component in FSSIM. Lund University.

Thornton P. K. 2010 Livestock production: recent trends, future prospects. Phil. Trans. R. Soc. B 365, 2853–2867.

United Nations, 2010. On-Line Database: Population.

US EPA, nd. SUSTAINABLE FOOD WASTE MANAGEMENT THROUGH THE FOOD RECOVERY CHALLENGE: Feed People Not Landfills.

Western Australia Dept of Agriculture, 2002. Guidelines for the Environmental Management of Beef Cattle Feedlots in Western Australia. Department of Agriculture.

White, R.P., Murray, S., Rohweder, M., Prince, S.D., Thompson, K.M.J., 2000. Grassland ecosystems. World Resources Institute.

Zhang, R., El-Mashad, H., Hartman, K., Wang, F., Rapport, J., Choate, C., Gamble, P., 2005. Anaerobic Phased Solids Digester Pilot Demonstration Project: Characterization of Food and Green Wastes As Feedstock for Anaerobic Digesters (CONTRACTOR REPORT). California Energy Commission.

Zia, U.E., Mahmood, T., Ali, M.R., 2011. Dairy Development in Pakistan. FOOD AND AGRICULTURE ORGANIZATION OF THE UNITED NATIONS FAO, Rome.
